# Analysis of Genetic Diversity and Population Structure of *Orobanche foetida* Populations From Tunisia Using RADseq

**DOI:** 10.3389/fpls.2021.618245

**Published:** 2021-04-13

**Authors:** Amal Boukteb, Shota Sakaguchi, Yasunori Ichihashi, Mohamed Kharrat, Atsushi J. Nagano, Ken Shirasu, Mariem Bouhadida

**Affiliations:** ^1^Faculty of Science of Tunis, University of Tunis El Manar, Tunis, Tunisia; ^2^Field Crop Laboratory, National Institute of Agricultural Research of Tunisia, Carthage University, Tunis, Tunisia; ^3^Graduate School of Human and Environmental Studies, Kyoto University, Kyoto, Japan; ^4^RIKEN BioResource Research Center, Tsukuba, Japan; ^5^Faculty of Agriculture, Ryukoku University, Otsu, Japan; ^6^RIKEN Center for Sustainable Resource Science, Yokohama, Japan

**Keywords:** parasitic plant, *Orobanche foetida*, RADseq, genetic diversity, population structure, Tunisia

## Abstract

*Orobanche foetida* Poiret is a holoparasitic plant that lacks chlorophyll and totally depending on its host for its growth. *Orobanche foetida* parasitizes host plant roots and extract nutrient and water via a haustorium. Although *O. foetida* distributes in the Mediterranean region as a wild plant parasite, it parasitizes faba bean causing serious damages which may reach 90% yield losses in Tunisia. Analysis of genetic diversity of the parasite is important to better understand its evolution and spread, remained largely unknown. In this work, we present the first study on genetic diversity and population structure using the robust technique Restriction-site-Associated DNA sequencing (RADseq) for *Orobanche* spp. We collected 244 samples of *O. foetida* from 18 faba bean fields in the north of Tunisia including 17 populations from the north-west and one population form the north-east. To overcome the difficulty of SNP discovery in *O. foetida* genome as a non-model and tetraploid plant, we utilized three different informatics pipelines, namely UNEAK, pyRAD and Stacks. This study showed that genetic differentiation occurred in the Tunisian *O. foetida* emphasizing the isolation by distance effect. However, no strong population clustering was detected in this work basing on the three data sets and clustering methods used. The present study shed the light on the current distribution and the genetic variation situation of the fetid broomrape in Tunisia, highlighting the importance of understanding the evolution of this parasite and its genetic background. This will aid in developing efficient strategies to control this parasite and its expansion in Tunisia and worldwide.

## Introduction

*Orobanche* is the largest genus among the holoparasitic members of *Orobanchaceae* with about 170 species (Uhlich et al., 1995). *Orobanche foetida* Poiret (common name: fetid broomrape) is one of the most virulent species of *Orobanche*; it is an obligate holoparasite, devoid of chlorophyll, parasitizing plant roots and totally depending on its host for its growth. It establishes a connection at host root level via a haustorium to extract nutrient and water. Poiret described the fetid broomrape for the first time in North Africa during his surveys on 1783 (Poiret, 1922). It is a tetraploid plant (2n = 4x = 76) (Román et al., 2003; Schneeweiss et al., 2004) characterized by a red color and a fetid smell. It was generally considered as a wild plant parasite widespread in natural habitats in the western Mediterranean countries; Tunisia, Algeria, Morocco, Spain and Portugal (Pujadas-Salvá et al., 2003).

In Tunisia, two *Orobanche* species were noticed by Chabrolin since 1939 (Chabrolin, 1939) on faba bean field, *O. foetida* Poir. and *O. crenata* Forsk (called at that time *O. speciosa*). At that period, the main constraint on faba bean crop in Tunisia was caused by *O. crenata*, while *O. foetida* was observed with insignificant damage. Currently, *O. foetida* is representing a real threat causing severe damages in Tunisia mainly on faba bean. In fact, *O. foetida* populations are more aggressive on faba bean than on other food legume crops (Kharrat and Halila, 1994). Whereas, in Spain and Portugal *O. foetida* was only observed on wild legumes, while *O. crenata* was highly damaging faba bean fields (Román et al., 2007b). Moreover, *O. foetida* in Morocco, was detected only on wild legumes such as *Scorpiurus* and on only one cultivated species *Vicia sativa* (common vetch), but never on faba bean, even when this legume crop was in close proximity, or even in the same *O. foetida*-infested field (Rubiales et al., 2005). Thus, the damages of *O. foetida* on faba bean have been reported only in Tunisia according to all researches conducted till now on this parasite.

Regarding the geographical distributions of *O. crenata* and *O. foetida* in Tunisia, differences have been reported. *Orobanche crenata* is observed mainly in the northern and central-eastern regions, whereas *O. foetida* is reported in the central and western north of Tunisia (Kharrat et al., 1992).

The potential of *O. foetida* to become a real threat to agriculture exists and has to be taken into account. Indeed, *O. foetida* may display a rapid host-differentiation, shifting from wild to cultivated legume hosts, as the case cited by Vaz Patto et al. (2008) in Morocco. Moreover, the increasing infestation of legume growing area with *O. foetida* in Tunisia is also of great interest from an evolutionary aspect, since this species is especifically parasitizing wild hosts in the other Mediterranean countries as mentioned above. Indeed, the fetid broomrape was described as a real threat on faba bean crop for the first time on 1992 in the north-west of Tunisia (Kharrat et al., 1992), causing severe damages with yield losses more than 90% (Abbes et al., 2007a) and ever since, *O. foetida* was detected on different crops such as chickpea (*Cicer arietinum*), grass pea (*Lathyrus sativus*), lentil (*Lens culinarus*), vetch (*Vicia sativa*) and fenugreek (*Trigonella foenum-graecum*) (Kharrat, 2002; Amri et al., 2009). The long-term persistence of *O. foetida* seeds in the field have impelled farmers to renounce to faba bean, the most grown pulse in Tunisia, thus, having a negative impact on soil fertility and farmer benefit.

Various methods were developed and adopted to control the parasitic plant at different stages. Some methods were adopted to reduce the parasitic seed production and its dispersal mainly via hand weeding, transplanting and deep sowing or by enhancing chemical soil fertility (Goldwasser and Rodenburg, 2013). Nevertheless, it is not possible to completely eliminate the parasite seed bank in the soil (Goldwasser and Rodenburg, 2013). Other methods had to deal with the existing seed bank using solarization method and cropping system (Trap crops ‘false host’, Catch crops and Crop rotations) (Abebe et al., 2005; Goldwasser and Rodenburg, 2013; Abbes et al., 2019). Chemical control was also adopted and the best result for *O. foetida* was obtained by the application of Glyphosate at budding stage and 15 days later (Kharrat and Halila, 1996). However, this herbicide suppress phytoalexin biosynthesis in legumes (Lévesque and Rahe, 1992; Sharon et al., 1992), which affects the crop immunity system leading to diseases (Foy et al., 1989). Additionally, biological control approaches were conducted mainly, using insects such as *Phytomyza orobanchia* for broomrape (Linke et al., 1990; Klein and Kroschel, 2002) and fungal pathogens such as *Fusarium* sp. for *O. foetida* and *O. crenata*, which decreased the parasite emergence and biomass about 70-100% (Boutiti et al., 2008). All the above methods revealed to be well-functioning in some cases, but approving an integrate strategy using several methods in the same time showed to be more efficient. Improving the genetic capacity of the plant host to deal with the parasite attacks through breeding for resistance, seems to be the most appropriate and cost-effective means of managing. Many progresses have already been achieved in this topic. Cubero and Moreno (1999) reported that the broomrape resistance is of polygenic nature and controlled by a quantitative genetic system with strong additive effects. Studies of quantitative trait loci (QTL) for *O. crenata* resistance in legumes using molecular markers have strengthen its complex inheritance (Román et al., 2002; Valderrama et al., 2004). Moreover, the aggressiveness of different broomrape biotypes is highly variable. Thus, better understanding of the evolution of *Orobanche* is primordial to better estimate their aggressiveness and their capacity to attack other crops. Indeed, several sources of resistance to broomrape mainly for *O. crenata* from Egypt (Giza 402), Spain (VF172), ICARDA (BPL2210) were used in crosses to release new faba bean varieties with good tolerance to *O. crenata* and *O. foetida* such as Giza 429, Giza 674, Giza 843, etc. from Egyptian breeding programs (El-Shirbini and Mamdouh, 2004); Baraca, Alameda, Brocal, Quijote, and Navio from Spain (Cubero et al., 1992; Nadal et al., 2004; Rubiales et al., 2014). In Tunisia, the faba bean breeding program released two varieties “Najeh” (Kharrat et al., 2010) and “Chourouk” (Amri et al., 2019) tolerant to *O. foetida* and *O. crenata* (Abbes et al., 2007b) from the different sources of resistance cited above and used in crosses as genitors with local lines selected for their agronomic performances.

In order to establish efficient strategies in Breeding programs, it is required to investigate the genetic variability within and among *O. foetida* populations. Genetic diversity studies gives a wise information about the target populations particularly on their appartenance to one or many gene pools. In fact, several studies were conducted to analyze the genetic diversity in *Orobanche* genera for several species using different techniques mainly; isoenzymes for *O. crenata* (Verkleij et al., 1986) and *O. cumana* (Castejon Munoz et al., 1991), RAPD for *O. crenata* (Román et al., 2001b), *O. cumana* (Gagne et al., 1998), *O. gracilis* (Román et al., 2007a) and *O. ramosa* (Brault et al., 2007), ISSR for *O. minor* (Westwood and Fagg, 2004), AFLP for *O. cumana* (Gagne et al., 2000). Ever since, specific markers were developed for some species such SSR for *O. cumana* by Pineda-Martos et al. (2014b) which offers the opportunity to study *O. cumana* diversity in many countries; Spain (Pineda-Martos et al., 2013; Martín-Sanz et al., 2016), Tunisia (Jebri et al., 2017) and Turkey (Bilgen et al., 2019). These markers were also used recently to study the genetic diversity of *O. crenata* populations from Ethiopia (Belay et al., 2020). Besides, SRAP markers were used to study *O. crenata* populations from Morocco (Ennami et al., 2017). Regarding *O. foetida*, the first study was performed using RAPD markers (Román et al., 2001a) focusing on Tunisian and Spanish populations, later AFLP markers were used to study the genetic diversity of Moroccan populations (Vaz Patto et al., 2008) of *O. foetida* collected from wild plants and one crop showing a genetic variation between them. Moreover, Bouhadida et al. (2015) studied the Tunisian populations of *O. foetida* collected from five legume crops using the universal markers RAPD. Thus far, no specific markers have been developed for *O. foetida*.

The ongoing progress of sequencing technologies offers the possibility to conduct advanced research on non-model plant. Restriction-site-Associated DNA sequencing (RADseq) (Davey and Blaxter, 2010) is a promising technique for population genetics particularly for species with limited genetic resources. Single nucleotide polymorphisms (SNPs) obtained by RADseq proved to be more informative than microsatellites in the matter of inferring population structure and relatedness estimation between individuals (Bruneaux et al., 2013; Thrasher et al., 2018; Lemopoulos et al., 2019). However, challenges arise for polyploidy species during data analysis in order to differentiate true alleles at a single locus from those generated by paralogs and duplicates (McMullen et al., 2009).

The purpose of the present study is to investigate genetic diversity and population structure of the Tunisian *O. foetida* populations collected from 18 natural infested faba bean fields using RADseq. To deal with the tricky SNP calling task, we used three different pipelines previously applied for polyploidy species; pyRAD (Eaton, 2014) a *de novo* assembler for RADseq loci, UNEAK developed especially for species without a reference genome (Lu et al., 2013) and Stacks (Catchen et al., 2013) which presumes the studied species as diploid.

## Materials and Methods

### Sample Collection

**FIGURE 1:**
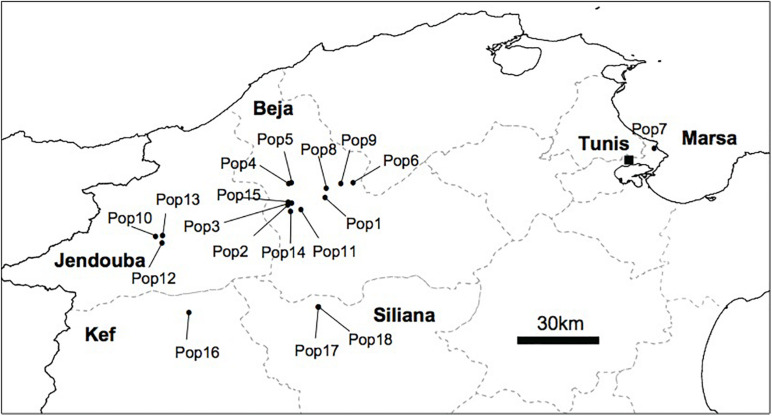
Geographic distribution of the 18 *O. foetida* collected from faba bean field in the North of Tunisia.

We conducted several surveys from 2010 to 2016 covering large areas in the north, the center and the south of Tunisia, known as habitats of different *Orobanche* species. In total, we sampled 18 populations of *O. foetida* from naturally infested faba bean field representing 244 samples of *O. foetida* plants from the north of Tunisia. The geographic location of the collected population is depicted in Figure 1.

### DNA Extraction, Library Prep and Sequencing

DNA was extracted from fresh floral buds (180 mg per sample) using innuPREP Plant DNA Kit (AnalytikJenaInd, Berlin, Germany) following the manufacturer’s instructions. Restriction-site-Associated DNA (RAD) libraries were prepared following the protocol according to Sakaguchi et al. (2015). In brief, we performed an enzymatic digestion using *Bgl*II and *Eco*RI and ligation with Y-shaped adaptors followed by a PCR with KAPA HiFi HS ReadyMix (KAPA BIOSYSTEMS). Size selection for fragments of approximately 350 base pairs (bp) was carried out using the E-Gel size select (Life technologies, CA, United States). We conducted a single-end sequencing with a read length of 51bp with the Illumina HiSeq 2500 platform (Illumina, CA, United States).

### Bioinformatic Analysis

The SNP calling was a challenging task due to the absence of a reference genome and the tetraploidy of *O. foetida*. We used three different software or workflows for comparison; (1) The Universal Network-Enabled Analysis Kit (UNEAK) implemented on TASSEL v3.0 software characterized by its ability to resolve the paralog, repeat issues and to analyze ploidy organisms (Lu et al., 2013), (2) Stacks, which distinguishes between sequence polymorphism and sequencing errors by the inclusion of a maximum likelihood statistical model (Catchen et al., 2013), and (3) pyRAD (Eaton, 2014), which allows a better identification of homology across highly divergent samples by the incorporation of indel variation (Qi et al., 2015).

We started our analysis by trimming raw reads with TRIMMOMATIC v.0.32 software (using the commands LEADING:19, TRAILING:19, SLIDINGWINDOW:30:20, AVGQUAL:20 and MINLEN:51). For the UNEAK pipeline, Tag counts were generated from FastQ files with the FastqToTagCountPlugin, then were merged with the MergeMultipleTagCountPlugin, the minimum count of a tag that must be present was defined to five. Tag pair was identified for SNP calling with UTagCountToTagPairPlugin and the error tolerance rate was specified as 0.03. Then, UTagPairToTBTPlugi and UTBTToMapInfoPlugin were applied before generating the HapMap file with UMapInfoToHapMapPlugin by fixing minimum and maximum allele frequency on mnMAF = 0.05 and –mxMAF = 0.5,–mnC = 0.5 and –mxC = 1. For Stacks, we set the minimum number of identical reads required to create a stack (*m* = 3), the nucleotide mismatches between loci within a single individual (*M* = 2), and the mismatches between loci when building the catalog (*n* = 1). The SNP genotype for each individual was exported with a minimum read depth of 6, using the ‘populations’ program implemented in Stacks. Finally, we set the following parameters for pyRAD workflow; 0.95 similarity, MinCov = 108, MaxSH p.50 and ploidy = 4.

### Population Analysis

To infer the genetic structure of studied populations, we used 4 different methods. Firstly, we used two Bayesian clustering programs relying on Markov Chain Monte Carlo algorithms (MCMC) to explore genetic structure in the genetic data; fastSTRUCTURE, a non-spatial method and TESS3 a spatial admixture analysis (Caye et al., 2016). Firstly, fastSTRUCTURE (Raj et al., 2014), can perform model-based inference of genetic ancestry by assuming random mating and linkage equilibrium within genetic clusters, and have been used for a broad range of sexually reproducing organisms (Falush et al., 2003; Harter et al., 2004). The model was set to allow admixture and correlated allele frequencies between genetic clusters, with an option of specifying sampled site (“LOCPRIOR” model). The 20 independent runs with 50000 burn-in and 100000 MCMCs were performed for each K value from 1 to 10 on a Linux workstation. Secondly, TESS3 provides ancestry coefficients estimations by combining matrix factorization and spatial statistical methods (Caye et al., 2016). It was developed in order to improve the inference of admixture proportions when they are variable across space (Durand et al., 2009). We applied a geno format file to run TESS3 in the Linux R environment according to the following parameters; K = 1:18, method = “projected.ls”, ploidy = 2, max.iteration = 1000, rep = 20, keep = “best.” The visualization of barplots was performed using the R package “Pophelper” v2.3.0 (Francis, 2017) in R v3.5.3 (R Core Team, 2019). To complement the fastSTRUCTURE and TESS3 analysis, we carried out a discriminant analysis of principal components (DAPC), which does not assume any population models using the “adegenet” v2.1.1 (Jombart and Ahmed, 2011) package in R v3.5.3 (R Core Team, 2019). Additionally, we performed the principal component analysis (PCA) using the “adegenet” v2.1.1 (Jombart and Ahmed, 2011) package in R v3.5.3 (R Core Team, 2019), to extract the synthetic variables in order to represent major genetic variations.

Genetic diversity and genetic variance were estimated within and among the 18 collected populations. We used “hierfstat” v0.04-22 package (De Meeûs and Goudet, 2007) in the R v3.5.3 (R Core Team, 2019) to determine genetic diversity parameters including the allelic richness (Ar), the observed heterozygosity (*H*o), the expected heterozygosity (*H*e), the inbreeding coefficient (*F*_*IS*_) and the fixation index (*F*_*ST*_). The analysis of molecular variance (AMOVA) was carried out to examine patterns of genetic variation and to estimate variance components for the 18 collected populations using “poppr” v2.8.2 (Kamvar et al., 2014) package in R v3.5.3 (R Core Team, 2019) (using poppr.amova function). Furthermore, we tested the isolation by distance (IBD) by performing a Mantel test for correlation between Edwards’ distances and Euclidean geographic distances for the 18 collected populations with “adegenet” v2.1.1 (Jombart and Ahmed, 2011) package in R v3.5.3 (R Core Team, 2019) (using mantel.randtest function) by conducting 999 permutations for significance assessment.

## Results

### Population Structure

**FIGURE 2:**
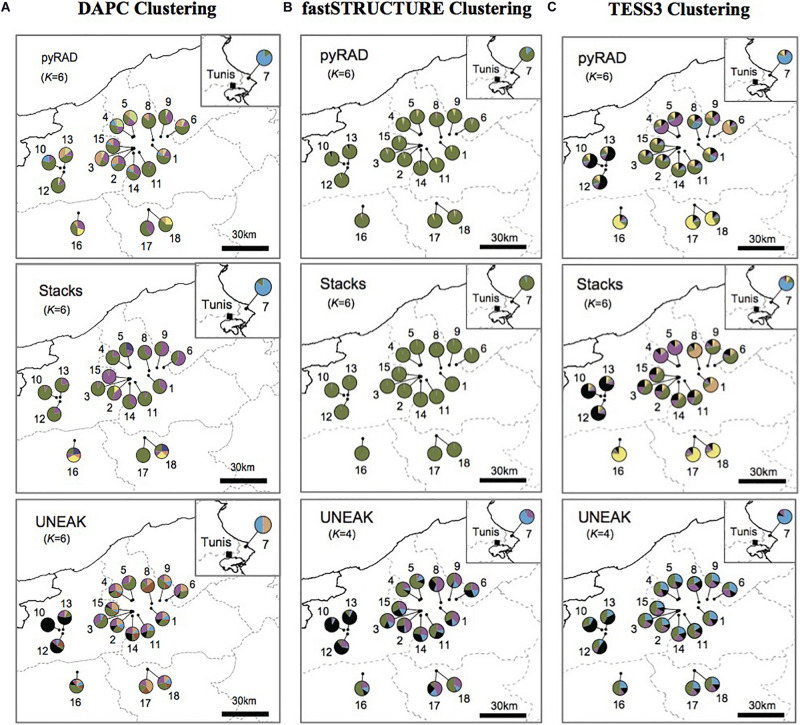
The average membership probabilities of each population (estimated Q values), inferred by **(A)** discriminant analysis of principal components (DAPC), **(B)** fastSTRUCTURE and **(C)** TESS3, as pie charts superimposed onto the location map. This is a comparison of the three used data sets: pyRAD, Stacks, and UNEAK.

According to our surveys conducted in several regions known as natural habitats for *Orobanche* spp., we detected the presence of *O. foetida* populations only in the north of Tunisia as mentioned in Figure 1. To investigate population structure of this fetid broomrape collected from the north of the country, we applied three different clustering methods including model-based methods (TESS3 and fastSTRUCTURE) and non-model-based method (DAPC) to the three genetic data sets generated by pyRAD, Stacks and UNEAK pipelines. For a better comparison among the clustering patterns, we superimposed the pie charts of the average membership probabilities of each population (estimated Q values) on geographic maps (Figure 2). We defined the parameter K, which describes the number of subpopulations that make up the total population (Verity and Nichols, 2016), as the minimum values of Ks that can capture biologically interpretable genetic structure (Pritchard et al., 2000). In fact, this criterion considers the appropriate Ks, after which, any increase in K contributes to a new cluster assigned to any population at very low proportion, while admitting other statistic methods (e.g., delta K method), they may indicate smaller values of Ks (Supplementary Figure 4). Thus, we adopted the above criterion since manual inspection of the clustering patterns for sequential Ks merits in grasping the effective genetic structure within the data sets. Overall, while we recognize certain levels of differences in genetic clustering, our comparison of clustering patterns revealed some populations that were differentiated from others across the genetic data and clustering methods. Pie charts represented in the Figure 2 with different color patterns were variable and pointed out the ancestral contribution in each studied population. It is important to mention the predominance of the light blue in population 7 (Pop7) (Figure 2). Moreover, the black was depicted mainly among Jendouba populations (Pops 10, 12, 13) in TESS3 (Figure 2C) using the three data set and in DAPC and fastSTRUCTURE using UNEAK data set (Figures 2A,B). Furthermore, the green was detected mainly among populations of Beja Governorate, with some differences in its proportion depending on the genetic data sets and the clustering methods. It’s necessary to highlight the grouping of populations 16, 17 and 18 using TESS3 [pyRAD (K = 6) and Stacks (K = 6) data sets] (Figure 2C) showing similar color patterns and the predominance of the yellow, however, DAPC and fastSTRUCTURE (Figures 2A,B) failed to detect any meaningful clusters with different frequencies among these populations using the same data sets. Eleven populations form Beja Governorate (Pops 1, 2, 3, 4, 5, 6, 8, 9, 11, 14, 15) showed similar color patterns using the several clustering methods through the three data set.

**FIGURE 3:**
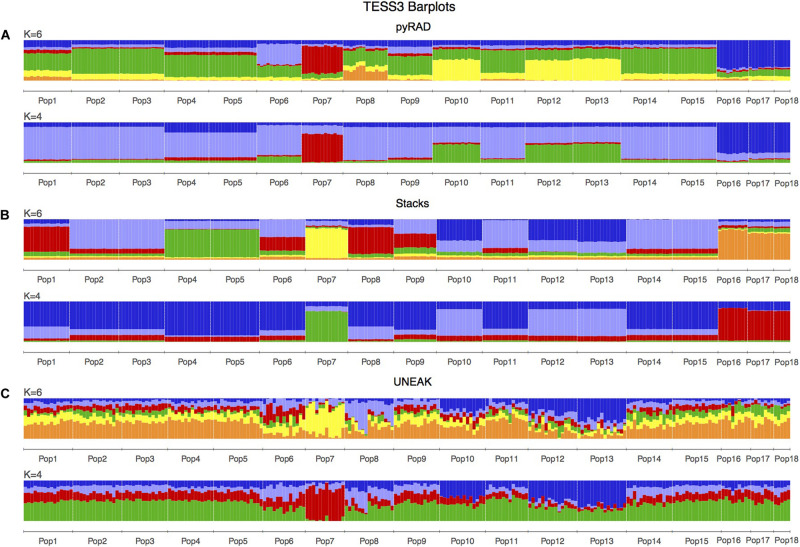
TESS3 clustering presented as barplots including 18 populations of *O. foetida*. This is a comparison of the three used data sets: **(A)** pyRAD, **(B)** Stacks and **(C)** UNEAK. The number of genetic clusters (K) are given above each barplot, while the populations are indicated in below of each barplot and divided by white lines.

**FIGURE 4:**
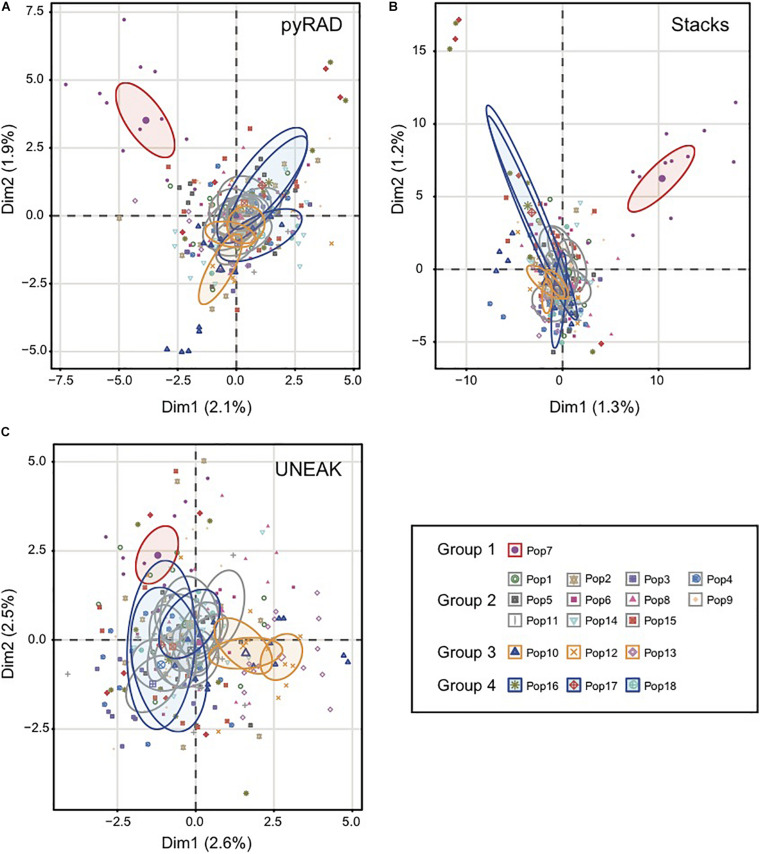
Principal coordinate analysis (PCA) based on SNP generated by the three data set; **(A)** pyRAD, **(B)** Stacks and **(C)** UNEAK. Colored ellipses showed the belonging of each population to one of the four detected groups (Group1:Red; Group 2:Gray; Group 3:Orange; Group 4:Blue). The ellipses represent 95% confidence range.

Basing on Figure 2, we noticed a differentiation among the collected populations indicating a potential grouping pattern. Thus, we suggested the distribution of the 18 populations accordingly in four groups as follows; Group 1 representing the population 7 collected from Tunis Governorate; Group 2 including 11 populations form Beja Governorate (Pops 1, 2, 3, 4, 5, 6, 8, 9, 11, 14, 15); Group 3 representing populations from Jendouba Governorate (Pops 10, 12, 13) and finally Group 4 composed by populations from Kef Governorate (Pop 17) and Siliana Governorate (Pops 17, 18). We used the clustering methods TESS3 and PCA for testing the robustness of population structure. Firstly, we presented the TESS3 clustering on barplot using the three data sets for K = 4 since we assumed four groups among the studied populations, and K = 6 in order to show further inferred clusters (Figure 3). PyRAD and Stacks data sets showed almost similar clustering patterns without confirming the existence of a strong genetic structure among the studied populations (Figures 3A,B). However, UNEAK did not show any clear clustering (Figure 3C). Secondly, for further investigation of the population grouping, we analyzed the PCA plots using the three data sets pyRAD, Stacks and UNEAK (Figure 4). To simplify the comparison among them, we used specific colors for ellipses for each potential group; red for Group1, gray for Group 2, orange for Group 3 and blue for Group 4. The divergence of the population 7 (Group 1, red), located at Tunis Governorate, from the others was clear using the three data sets and more emphasized with Stacks data set. Moreover, we recognized a slight divergence among the assessed groups. However, we observed an overlapping especially among the three groups representing populations from the north-west using the three data sets.

Additionally, we calculated the pairwise *F*_*ST*_ among the four groups in order to investigate the genetic distance among them. As expected, the highest *F*_*ST*_ values were observed between Group 1 (north-east) and the other Groups (north-west) using the three datasets (Table 1). Whereas, the lowest *F*_*ST*_ values were detected firstly between Group 2 and Group 3 using Stacks data set (*F*_*ST*_ = 0.0078), secondly, between Group 2 and Group 4 using pyRAD (*F*_*ST*_ = 0.0109) and UNEAK (*F*_*ST*_ = 0.0080) data sets as shown in Table 1. Overall, we detected a low *F*_*ST*_ values among the four potential groups using the three data sets.

**TABLE 1:** Pairwise Fixation index *F*_*ST*_ among the four assigned groups of the studied *O. foetida* populations using the three data sets; pyRAD, Stacks and UNEAK.

pyRAD				
	Group1	Group2	Group3	Group4
Group1	NA	0.0752	0.0867	0.0893
Group2	0.0752	NA	0.0123	**0.0109**
Group3	0.0867	0.0123	NA	0.0266
Group4	**0.0893**	0.0109	0.0266	NA

**Stacks**				

	**Group1**	**Group2**	**Group3**	**Group4**

Group1	NA	0.0491	0.0625	0.0585
Group2	0.0491	NA	**0.0078**	0.0141
Group3	**0.0625**	0.0078	NA	0.0203
Group4	0.0585	0.0141	0.0203	NA

**UNEAK**				

	**Group1**	**Group2**	**Group3**	**Group4**

Group1	NA	0.0406	0.0626	0.0490
Group2	0.0406	NA	0.0168	**0.0080**
Group3	**0.0626**	0.0168	NA	0.0312
Group4	0.0490	0.0080	0.0312	NA

**FIGURE 5:**
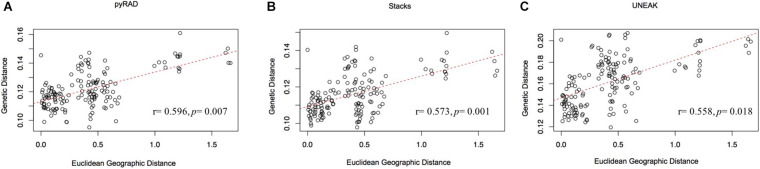
Mantel test for matrix correlation between Edwards’ distances and Euclidean geographic distances for 18 *O. foetida* populations using the three data sets: **(A)** pyRAD, **(B)** Stacks, and **(C)** UNEAK.

Furthermore, the IBD analysis (Figure 5) revealed significant and positive association between genetic and geographic distances among the 18 collected populations using the three data sets (UNEAK: Mantel’s r = 0.573, *p* = 0.001; Stacks: Mantel’s r = 0.558, *p* = 0.018; pyRAD: Mantel’s r = 0.596, *p* = 0.007).

### Genetic Diversity

The genetic diversity within and among populations was estimated basing on the 18 collected populations. According to Table 2, we observed that the variability parameters values (*A*r, *H*o, *H*e and *F*_*IS*_) are similar among the 18 populations for each data set. Although, differences arise when comparing these parameters among the three data sets. However, UNEAK data set revealed higher values compared to those from pyRAD and Stacks data sets. Moreover, the mean values of allelic richness (*A*r) among the 18 populations were similar using pyRAD and Stacks data sets (1.06), while it was slightly higher using UNEAK data set (1.28). Furthermore, the observed heterozygosity (*H*o) was lower than the expected heterozygosity (*H*e) for the three data sets excepting for Pops 6, 8, 12, 13 using UNEAK data set and Pop 13 using Stacks data set. The *H*o values were ranged from 0.192 to 0.348 with a mean value of 0.253 for UNEAK data set, from 0.044 to 0.059 with a mean value of 0.048 for Stacks data set and varied from 0.032 to 0.046 with a mean value of 0.039 for pyRAD data set. While the *H*e mean values were 0.28, 0.062, and 0.058 for UNEAK, Stacks and pyRAD, respectively. Regarding the inbreeding coefficient (F_*IS*_), UNEAK data set generated F_*IS*_ values ranged from −0.131 to 0.240 (mean value = 0.097), while for Stacks, F_*IS*_ values were comprised between −0.017 and 0.259 (mean value = 0.166) and they were ranged from 0.092 to 0.279 (mean value = 0.208) for pyRAD data set.

**TABLE 2:** Summary of genetic variability parameters at the populations level using the three data sets; pyRAD, Stacks, and UNEAK.

	*A*r (Mean ± SD)			*H*o (Mean ± SD)			*H*e (Mean ± SD)			*F*is (Mean ± SD)		
Populations	pyRAD	Stacks	UNEAK	pyRAD	Stacks	UNEAK	pyRAD	Stacks	UNEAK	pyRAD	Stacks	UNEAK
Pop1	1.06 (0.117)	1.06 (0.127)	1.27 (0.179)	0.037 (0.002)	0.047 (0.001)	0.205 (0.015)	0.058 (0.003)	0.061 (0.001)	0.271 (0.013)	0.246 (0.020)	0.156 (0.008)	0.240 (0.033)
Pop2	1.06 (0.120)	1.06 (0.128)	1.28 (0.178)	0.038 (0.002)	0.044 (0.001)	0.236 (0.015)	0.059 (0.003)	0.061 (0.001)	0.279 (0.012)	0.260 (0.021)	0.209 (0.008)	0.149 (0.031)
Pop3	1.06 (0.116)	1.06 (0.126)	1.27 (0.172)	0.035 (0.002)	0.044 (0.001)	0.220 (0.016)	0.058 (0.003)	0.061 (0.001)	0.268 (0.012)	0.249 (0.020)	0.206 (0.008)	0.181 (0.034)
Pop4	1.05 (0.122)	1.06 (0.131)	1.25 (0.189)	0.032 (0.002)	0.040 (0.001)	0.192 (0.015)	0.056 (0.003)	0.062 (0.001)	0.250 (0.013)	0.279 (0.021)	0.259 (0.009)	0.214 (0.035)
Pop5	1.06 (0.121)	1.06 (0.128)	1.27 (0.174)	0.039 (0.002)	0.045 (0.001)	0.228 (0.015)	0.062 (0.003)	0.063 (0.001)	0.277 (0.012)	0.252 (0.018)	0.211 (0.008)	0.170 (0.031)
Pop6	1.06 (0.115)	1.06 (0.133)	1.30 (0.158)	0.045 (0.003)	0.052 (0.001)	0.326 (0.017)	0.058 (0.003)	0.066 (0.001)	0.300 (0.011)	0.159 (0.017)	0.151 (0.008)	−0.039 (0.028)
Pop7	1.06 (0.128)	1.06 (0.135)	1.26 (0.203)	0.040 (0.002)	0.047 (0.001)	0.244 (0.018)	0.061 (0.003)	0.065 (0.001)	0.261 (0.014)	0.212 (0.020)	0.198 (0.008)	0.073 (0.036)
Pop8	1.05 (0.119)	1.06 (0.128)	1.29 (0.182)	0.046 (0.003)	0.056 (0.001)	0.330 (0.018)	0.054 (0.003)	0.059 (0.001)	0.289 (0.013)	0.101 (0.017)	0.046 (0.007)	−0.089 (0.026)
Pop9	1.06 (0.123)	1.06 (0.131)	1.29 (0.176)	0.038 (0.002)	0.045 (0.001)	0.235 (0.015)	0.060 (0.003)	0.063 (0.001)	0.293 (0.012)	0.264 (0.021)	0.219 (0.009)	0.196 (0.033)
Pop10	1.06 (0.127)	1.06 (0.132)	1.27 (0.181)	0.037 (0.002)	0.046 (0.001)	0.238 (0.015)	0.060 (0.003)	0.061 (0.001)	0.270 (0.013)	0.259 (0.022)	0.179 (0.008)	0.112 (0.030)
Pop11	1.05 (0.129)	1.06 (0.135)	1.27 (0.177)	0.036 (0.003)	0.043 (0.001)	0.204 (0.014)	0.058 (0.004)	0.060 (0.001)	0.272 (0.013)	0.238 (0.024)	0.180 (0.010)	0.236 (0.034)
Pop12	1.06 (0.117)	1.06 (0.126)	1.31 (0.155)	0.042 (0.002)	0.053 (0.001)	0.330 (0.017)	0.057 (0.003)	0.058 (0.001)	0.308 (0.011)	0.156 (0.018)	0.070 (0.007)	−0.018 (0.029)
Pop13	1.05 (0.120)	1.06 (0.127)	1.29 (0.186)	0.045 (0.003)	0.059 (0.001)	0.348 (0.020)	0.054 (0.003)	0.056 (0.001)	0.289 (0.013)	0.092 (0.018)	−0.017 (0.007)	−0.131 (0.030)
Pop14	1.06 (0.114)	1.06 (0.126)	1.29 (0.186)	0.041 (0.002)	0.050 (0.001)	0.348 (0.020)	0.057 (0.003)	0.059 (0.001)	0.289 (0.013)	0.187 (0.018)	0.123 (0.008)	−0.131 (0.030)
Pop15	1.06 (0.115)	1.06 (0.127)	1.29 (0.167)	0.038 (0.002)	0.045 (0.001)	0.293 (0.017)	0.058 (0.003)	0.061 (0.001)	0.286 (0.012)	0.228 (0.018)	0.194 (0.008)	0.017 (0.030)
Pop16	1.05 (0.127)	1.06 (0.140)	1.25 (0.198)	0.034 (0.002)	0.045 (0.001)	0.212 (0.016)	0.056 (0.003)	0.064 (0.001)	0.257 (0.014)	0.257 (0.025)	0.213 (0.010)	0.151 (0.038)
Pop17	1.05 (0.123)	1.06 (0.140)	1.25 (0.207)	0.036 (0.002)	0.046 (0.001)	0.229 (0.018)	0.057 (0.003)	0.064 (0.001)	0.258 (0.015)	0.225 (0.023)	0.195 (0.010)	0.077 (0.039)
Pop18	1.06 (0.148)	1.06 (0.163)	1.28 (0.218)	0.038 (0.003)	0.049 (0.001)	0.239 (0.019)	0.062 (0.004)	0.070 (0.002)	0.281 (0.016)	0.263 (0.030)	0.197 (0.013)	0.102 (0.043)

**A*r Allelic richness, *H*o observed heterozygosity, *H*e expected heterozygosity, inbreeding coefficient (*F*_*IS*_), standard error (SE).*

The AMOVA analysis revealed a significant level of genetic differentiation (Monte Carlo test *p* < 0.05) using the three data sets. At populations level, the molecular variance within populations was comprised between 96.29% and 96.74%, whereas among populations it varied from 3.25% to 3.70%.

## Discussion

In the present study, we used RADseq technique to investigate the genetic diversity and population structure of the Tunisian *O. foetida* biotype consisting of 244 samples collected from 18 different fields belonging to the main faba bean crop area in Tunisia. The population structure and the evaluation of genetic diversity of *O. foetida* represent the first step to understand the phylogenetic relationships within and among *Orobanche* populations, and to improve faba bean breeding program as well as pest management. Previously, three main genetic studies were conducted on *O. foetida* using dominant markers to evaluate the genetic diversity. Firstly, Román et al. (2007b), used 10 RAPD primers generating 168 polymorphic bands among 35 samples collected from legume crops in Tunisia. Secondly, Vaz Patto et al. (2008), studied five *O. foetida* populations from Morocco using three AFLP primers combinations where the proportion of polymorphic loci varied from 54.6% to 80%. Thirdly, Bouhadida et al. (2015), performed a genetic diversity study on Tunisian *O. foetida* populations (35 samples) collected from five crops (faba bean, chickpea, annual alfalfa, vetch and Narbonne vetch) using 10 RAPD primers, revealing 75 polymorphic bands. Due to the absence of specific markers and a reference genome for *O. foetida*, we chose the RADseq as a technique that did not require a gene set reference. To the best of our knowledge, this is the first study using RADseq technique to evaluate the genetic diversity of one of the most harmful *Orobanche* spp. in Tunisia, which represent a real threat worldwide.

### Comparison of RADseq Pipelines Used for SNP Calling

In the present study, we adopted a *de novo* assembly approach due to the absence of a reference genome, but the choice of the pipeline remains a relevant decision. Indeed, pipelines differ in the applied algorithms and the possibility to change some parameters. Besides for several pipelines, some intermediate files are not accessible; these files are temporary and deleted in the end of the analysis, which does not allow to track the evolution of the workflow and to detect some possible issues. For that purpose, we used three different software pipelines UNEAK, pyRAD and Stacks, which generate different numbers of SNP as expected.

The number of the detected SNPs varied among the three data sets. Indeed, Stacks generated the highest number (10,755 SNPs) followed by pyRAD (1,785 SNPs), however, UNEAK detected only 206 SNPs. This last result could be explained by the ability of UNEAK to eliminate paralogs, which are highly present in polyploidy species. Differences noticed in our results when applying different pipelines for the same dataset are also reported in various studies. In fact, the work of Torkamaneh et al. (2016) on the comparison of seven pipelines and two sequencing technologies, reported that UNEAK generated 82% more SNPs than Stacks. Moreover, Qi et al. (2015) found differences in phylogenetic patterns using pipelines requiring or not a reference genome (Stacks, TASSEL, UNEAK and pyRAD). Additionally, they obtained higher number of SNPs (25,000 to 54,000 SNPs) for reference genome pipelines than without a reference genome (13,000 to 24,000) which emphasizes the importance of a whole genome sequencing. In the present work, we performed a series of filtering of the generated SNP for the three data sets; nevertheless, we noticed a better population structure without filtering for Stacks and pyRAD. Therefore, we decided to maintain the filtering only for UNEAK data set since our main objective is to explore the population structure of the collected *O. foetida* populations.

Regarding the population structure analysis for the studied populations, the distribution and the number of the clusters revealed by the DAPC analysis varied among the three pipelines, but even that, a common cluster characterizing the population 7 was detected using the three data sets. TESS3 revealed a clear genetic structure using Stacks and pyRAD data sets comparing to fastSTRUCTURE which was not able to detect any meaningful clusters using these two data sets. Regarding UNEAK data set, we detected an informative clustering pattern only with fastSTRUCTURE. In fact, TESS3 was not able to detect a clear one.

### Population Structure

The population structure analysis was conducted using different clustering methods. Among them, TESS3 and fastSTRUCTURE, which are two Bayesian clustering models in population genetics that compute allele frequencies in each gene pool and estimate the proportions of an individual genome that originate from multiple ancestral gene pools (ancestry coefficients) (Frichot et al., 2014; Caye et al., 2016). In the present work, TESS3 outperformed fastSTRUCTURE by revealing clearer population structure pattern. This is due to the ability of TESS to provide better estimations of ancestry coefficients, when the levels of ancestral population divergence are low, by incorporating spatial arrangement of sampling localities in its analysis (Durand et al., 2009). Moreover, it has been already reported that the use of spatial models allowed detecting biologically meaningful clusters comparing to a non-spatial model such as STRUCTURE, which failed to detect any population structure especially when the ancestral level of differentiation was low (Barr et al., 2008; Lavandero et al., 2009; François and Durand, 2010). Indeed, basically according to the clustering maps presetting in Figure 2, we assigned the 18 collected populations into four groups, where three groups were assigned to a specific governorate (Tunis, Beja or Jendouba), while group 4 is representing a population from Kef governorate and two populations from Siliana governorate. The PCA plots, the DAPC (using pyRAD and Stacks data sets) and the fastSTRUCTURE (using only UNEAK data set) showed globally a similar clustering tendency. Nevertheless, all the methods used for population structure analysis in this study, TESS3 included, were unable to detect a strong population clustering. Additionally, the calculated pairwise *F*_*ST*_ among the four groups showed very low values in Table 1, which support the absence of a robust population clustering.

The population 7 (Group 1) from the north-east of Tunisia was the most genetically divergent one in comparison to the other three assigned groups representing populations from the north-west of the country, using the three data sets (UNEAK, pyRAD and Stacks), and four populations structure methods (TESS3, fastSTRUCTURE, DAPC, PCA). Additionally, the highest pairwise *F*_*ST*_ was observed between Group 1 and the other groups. This finding is consistent with a study on *Striga hermonthica* from Ethiopia (Welsh and Mohamed, 2011), in which populations located over 100 km from others were genetically divergent pointing out a comparable pattern with our data, since our population 7 is collected at 110-185 km from the other population locations. A similar situation of genetic separation between populations of Cuenca and Guadalquivir Valley has been reported for *O. cumana* in Spain by Pineda-Martos et al. (2013). Likewise, Westwood and Fagg (2004) reported two clearly different groups of *O. minor* populations in US, suggesting that they are developed from two distinct introduction events, as is probably the case for population 7 and the rest of populations of *O. foetida* studied in this present work. It is essential to report that the expanding urbanization and encroachment on rural areas in Tunis governorate these last decades significantly limited the faba bean cropping area, which affects the number of populations collected from this location of Tunisia (only population 7 from Tunis Governorate). Furthermore, about 60% of the collected populations are classified in Group 2 from Beja Governorate, known as the main growing area of faba bean in Tunisia which might reflect a narrow genetic background. In fact, the close geographic distance among these populations could be explained by the spread of high number of long-lived tiny seeds of *Orobanche* through this area. Indeed, seeds dissemination is highly facilitated by different factors as wind, machinery, cultivation practices, as well as the use of contaminated faba bean seed stocks (Satovic et al., 2009). All these factors may contribute to the expansion of Orobanche infestation through the faba bean growing area in Beja Governorate, showing a low genetic diversity and suggesting that they may belong to the same gene pool. Moreover, the genetic divergence of populations 10, 12 and 13 seems to be related to the geographic distance separating these populations from the others (located from 26 to 76 km from the other north-west populations). These observations are in concordance with the positive and significant correlation between the genetic and the geographic distance revealed by the IBD analysis. In fact, isolation by distance may play a key role in shaping the genetic variation among populations. Consistently, Unachukwu et al. (2017), reported that the genetic variation among the parasitic plant *Striga hermonthica* is monitored by geographic isolation.

### Genetic Diversity

In this work, we recorded genetic variability among the 18 populations of *O. foetida* in Tunisia according to the variability parameters Ar, *H*o, *H*e and *F*_*IS*_. These parameters were higher using UNEAK data set comparing to Stacks and pyRAD data sets. We recorded an average of 1.28 regarding the allelic richness for UNEAK data set and an average of 1.06 for Stacks and pyRAD data sets as reported in Table 2. Overall, the observed heterozygosity was lower than the expected heterozygosity among the three data set, which generate a positive *F*_*IS*_ value for the majority of the studied populations indicating an inbreeding behavior of the *O. foetida* populations in Tunisia. Similar results were also recorded among *O. cumana* populations from Turkey (Bilgen et al., 2019), Spain and Bulgaria (Pineda-Martos et al., 2013, 2014a) using SSR markers, pointing out a deviation from Hardy-Weinberg equilibrium. Furthermore, studies conducted on *Orobanche* spp. using co-dominant markers such as SSR markers on *O. cuman*a, allowed to figure out these parameters. Indeed, Genetic variation of *O. cumana* populations from Spain was investigated using 15 SSR markers, the variability parameters were averaged as follows; Ae (1.6), *H*o (0.04), and *H*e (0.33) (Pineda-Martos et al., 2013). These values are similar to our average values using UNEAK pipeline (Ae 1.28), *H*o (0.25) and *H*e (0.277), this could be explained by the co-dominance nature of markers used in both studies. Nevertheless, comparison among diversity studies is more conclusive using the same species and the same type of markers. Indeed, Bilgen et al. (2019) used 8 SSR markers to study *O. cumana* populations from Turkey. They reported a variation regarding mean values of *N*e (1.67), *H*o (0.21), *H*e (0.34) and the Fixation index (*F*) (0.34) and were able to compare their results with those of Pineda-Martos et al. (2013) in terms of heterozygosity levels in populations of *O. cumana* from Spain, Bulgaria and Turkey. For the case of *O. foetida*, up to now, few genetic diversity researches were performed, and the molecular markers used were dominant such as RAPD and AFLP markers (Román et al., 2007a; Vaz Patto et al., 2008; Bouhadida et al., 2015). These kinds of markers are not enough informative and did not allow to compute all the variability parameters cited above, thus, preventing a robust analysis of genetic diversity among populations and an accurate comparisons among diversity studies on *O. foetida*.

The results of AMOVA analysis were consistent among the three data sets showing a high genetic variation within the 18 populations (from 96.29% to 96.74%) while the remaining part was assigned to interpopulation variation. Consistently, in the study of Vaz Patto et al. (2008) using AFLP for *O. foetida* populations parasitizing wild plants and crop in Morocco, 86.63% of the variation was due to within-population variations, whereas only 13.75% was due to among-population variations. Moreover, in a study performed on fetid broomrape populations using RAPD markers by Román et al. (2001a), reported that 75.4% of the variation was due to within-population variations for Tunisian populations collected on legume crops (faba bean and chickpea).

The high genetic variation observed within populations lead us to consider that *O. foetida* may be an autogamous species. Furthermore, Román et al. (2001a) also suggested an autogamous behavior for Tunisian crop-infected populations and an allogamous behavior for Spanish wild-infected populations based on the genetic variation analysis. Similarly, difference between wild and weedy populations was also detected among the autogamous *Striga gesnerioides* populations parasitizing the wild legume *Indigofera hirsuta* L. and cultivated cowpea (Botanga and Timko, 2006). By contrast, no difference was detected between *O. cumana* populations growing on sunflower and those on wild plants, suggesting a potential gene flow between these populations in Bulgaria (Pineda-Martos et al., 2014a). According to Vaz Patto et al. (2008) reports, no clear separation was detected basing on cluster analysis between *O. foetida* population infecting common vetch and the population infecting wild legumes in Morocco. Furthermore, it is important to point out that the sampled populations in Morocco were collected from a predominant faba bean growing area where *O. foetida* was only detected on common vetch (Vaz Patto et al., 2008), while in Tunisia the major host of *O. foetida* is faba bean. This may be explained by host differentiation effect previously reported by Román et al. (2007b) between *O. foetida* populations infecting chickpea and faba bean in Tunisia. Moreover, the Moroccan *O. foetida* populations are all considered as *O. foetida* var. *broteri* (Vaz Patto et al., 2008) while populations used in our study are considered as *O. foetida Poir*. (Domina G, pers. Comm.). In the same context, Thorogood et al. (2008) observed a variability between *O. minor* var. *minor* and *O. minor* spp. *Maritiama* which were classified in distinct clusters using SCAR markers.

The attack of *O. foetida* on legumes and wild relatives was assumed for several years as an adaptation event (Román et al., 2007a; Vaz Patto et al., 2008; Satovic et al., 2009) while it was recorded as a crop parasite in Tunisia since 1905 on *Medicago truncatula* (Boeuf, 1905). Presumably, during the double infection of faba bean in Tunisia by *O. foetida* and *O. crenata* (Chabrolin, 1939), the fetid broomrape succeeded to eliminate *O. crenata*, due to the fact that, as an autogamous species it has a higher chance to survive and maintain than an allogamous one (Román et al., 2001a). This may explain the abundance of *O. foetida* and the absence of *O. crenata* in the main faba bean growing area in the north-west of Tunisia. Moreover, according to the literature, *O. foetida* was recorded attacking wild legumes since 1896 in the north-east of Tunisia (Bonnet and Barratte, 1896), but currently this species is totally absent in this area in Tunisia. Román et al. (2001a) supposed that *O. foetida* populations infecting wild plants are allogamous, and due to the growth of urbanization, these populations could not persist, and its presence has been decreased through time, which may explain the absence of this species in the north-east of Tunisia during this decade. Furthermore, as suggested by Vaz Patto et al. (2008), the *O. foetida* population infecting wild plants may give rise to new populations able to highly damage cultivated species as vetch in Morocco, faba bean in Tunisia and may affect other legume crops such as chickpea.

This present study traces the real situation of distribution of *O. foetida* populations in Tunisia and could be a valuable reference for the upcoming research projects focusing on *O. foetida*. Indeed, further research should be undertaken to explore different ecotypes of the fetid broomrape in the Mediterranean area with the perspective to understand the evolution of this parasite. Additionally, the evaluation of the parasitic potential of the studied populations should be carried out in order to analyze a possible correlation with their genetic diversity. On a wider level, a whole genome and transcriptome sequencing of *O. foetida*, as seen for *Striga asiatica* (Yoshida et al., 2019), will be of interest to better understand the genetic background allowing this species to overcome host defense mechanisms and became a serious threat to agriculture. Indeed, for a longtime, the damage caused by *O. foetida* on faba bean was limited in Tunisia until 1992 (Kharrat et al., 1992). Currently, *O. foetida* is considered as a real risk for faba bean but also start being for other legume crops such as chickpea. With this in prospect, it would be recommendable to prepare new efficient strategies to anticipate the potential danger of this parasite for several crop legumes in Tunisia and worldwide.

## Data Availability Statement

The original contributions presented in the study are publicly available. This data can be found here: NCBI’s Sequence Read Archive (Accession: PRJNA719284).

## Author Contributions

AB, YI, MB, and KS conceived and designed the experiments. AB, YI, and AN performed the experiments. AB and SS analyzed the data. AB, YI, SS, and MB wrote the manuscript. All authors contributed to the article and approved the submitted version.

## Conflict of Interest

The authors declare that the research was conducted in the absence of any commercial or financial relationships that could be construed as a potential conflict of interest.
