# Supplementary material for: Analysis of Genetic Diversity and Population Structure of Orobanche foetida Populations From Tunisia Using RADseq
Source: Front Plant Sci. 2021 Apr 13;12:618245. doi: 10.3389/fpls.2021.618245 (PMC8078179; doi:10.3389/fpls.2021.618245)
Supplement: Supplementary file 1 [file Data_Sheet_1.docx]

***Supplementary Material***


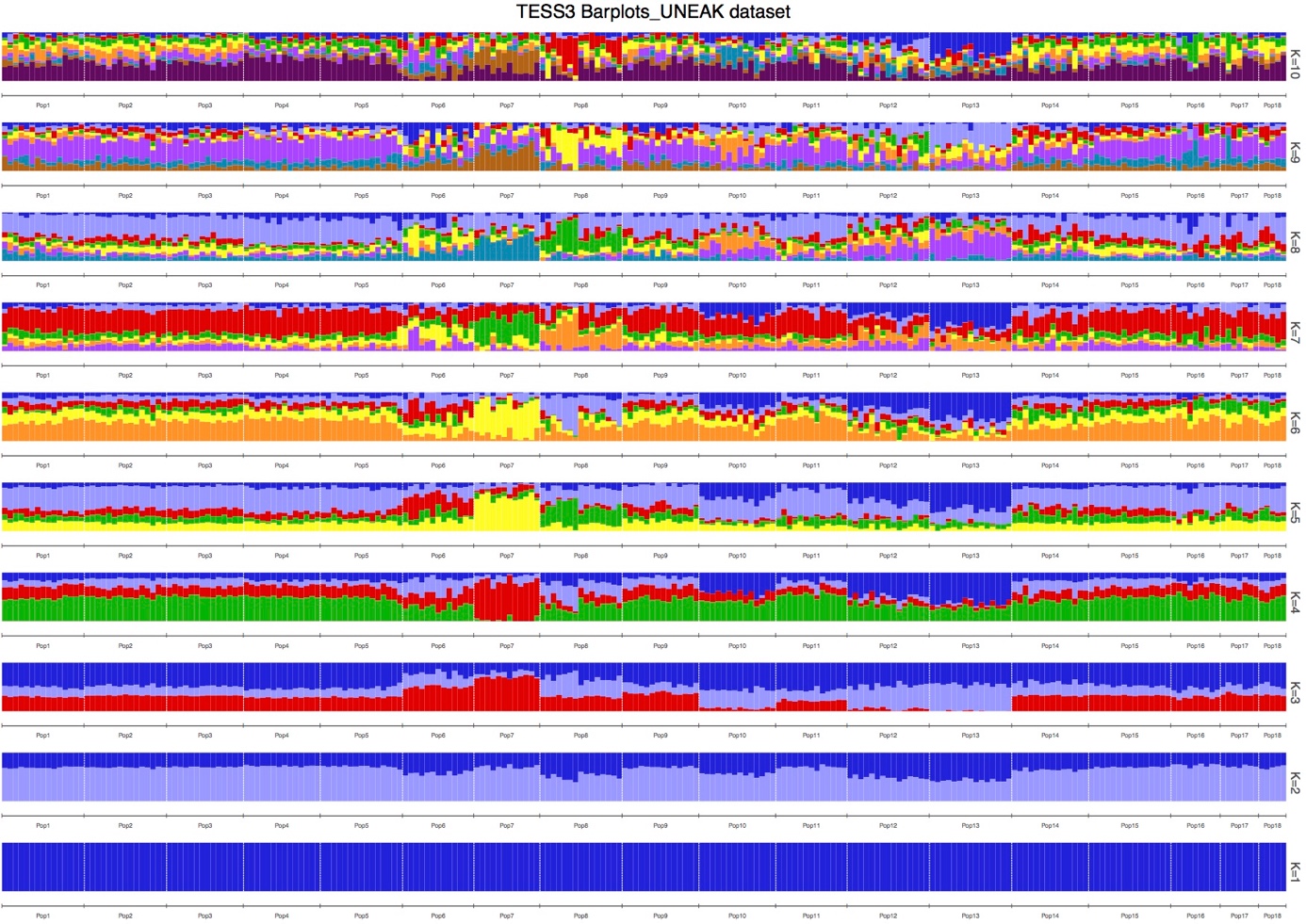


**Figure S1.** TESS3 clustering presented as barplots including 18 populations of *O. foetida* using UNEAK data set. The number of genetic clusters (K) are given on the right of each barplot, while the populations are indicated in below of each barplot and divided by white lines. (K=1 to K=10).


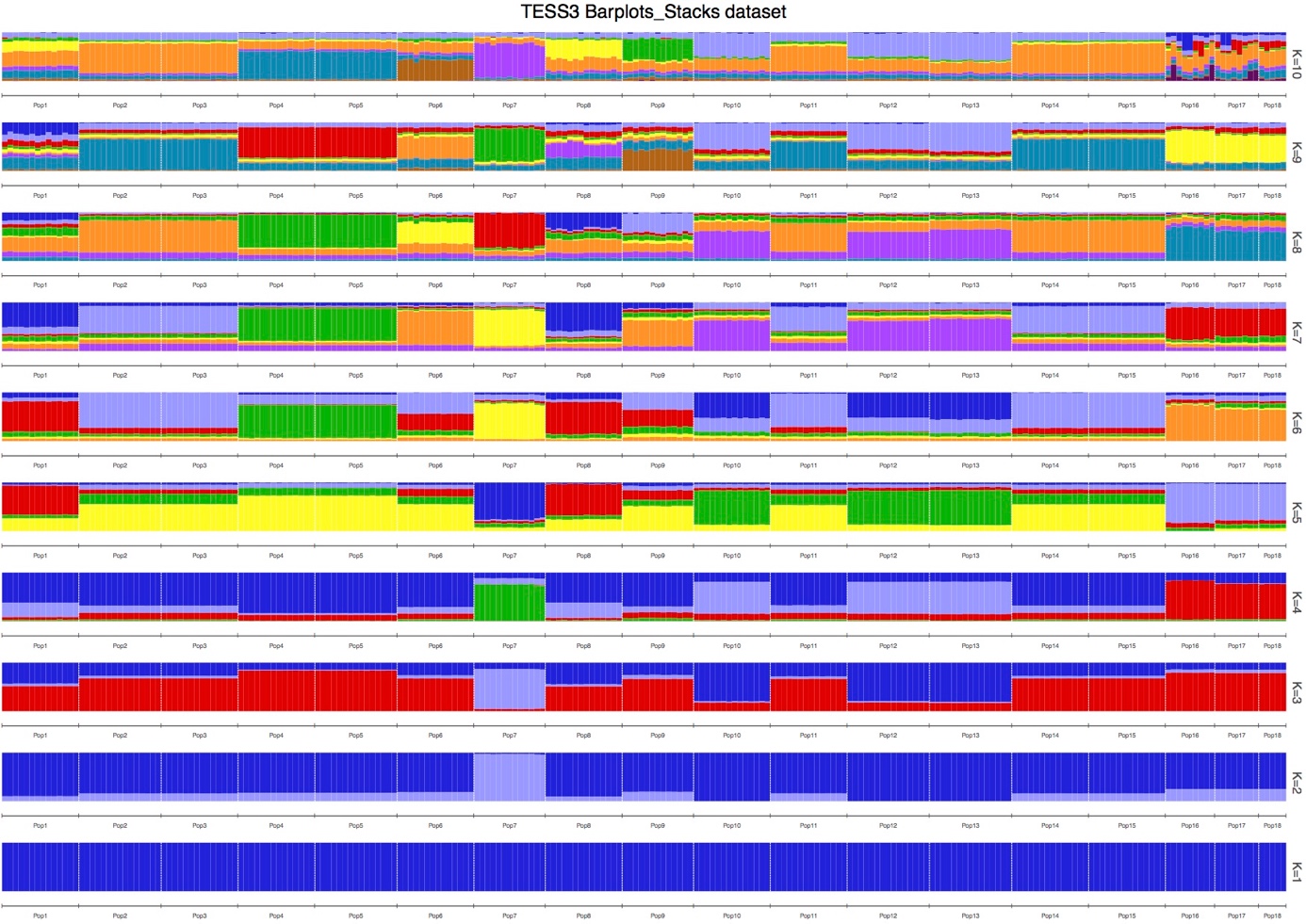


**Figure S2.** TESS3 clustering presented as barplots including 18 populations of *O. foetida* using Stacks data set. The number of genetic clusters (K) are given on the right of each barplot, while the populations are indicated in below of each barplot and divided by white lines. (K=1 to K=10).


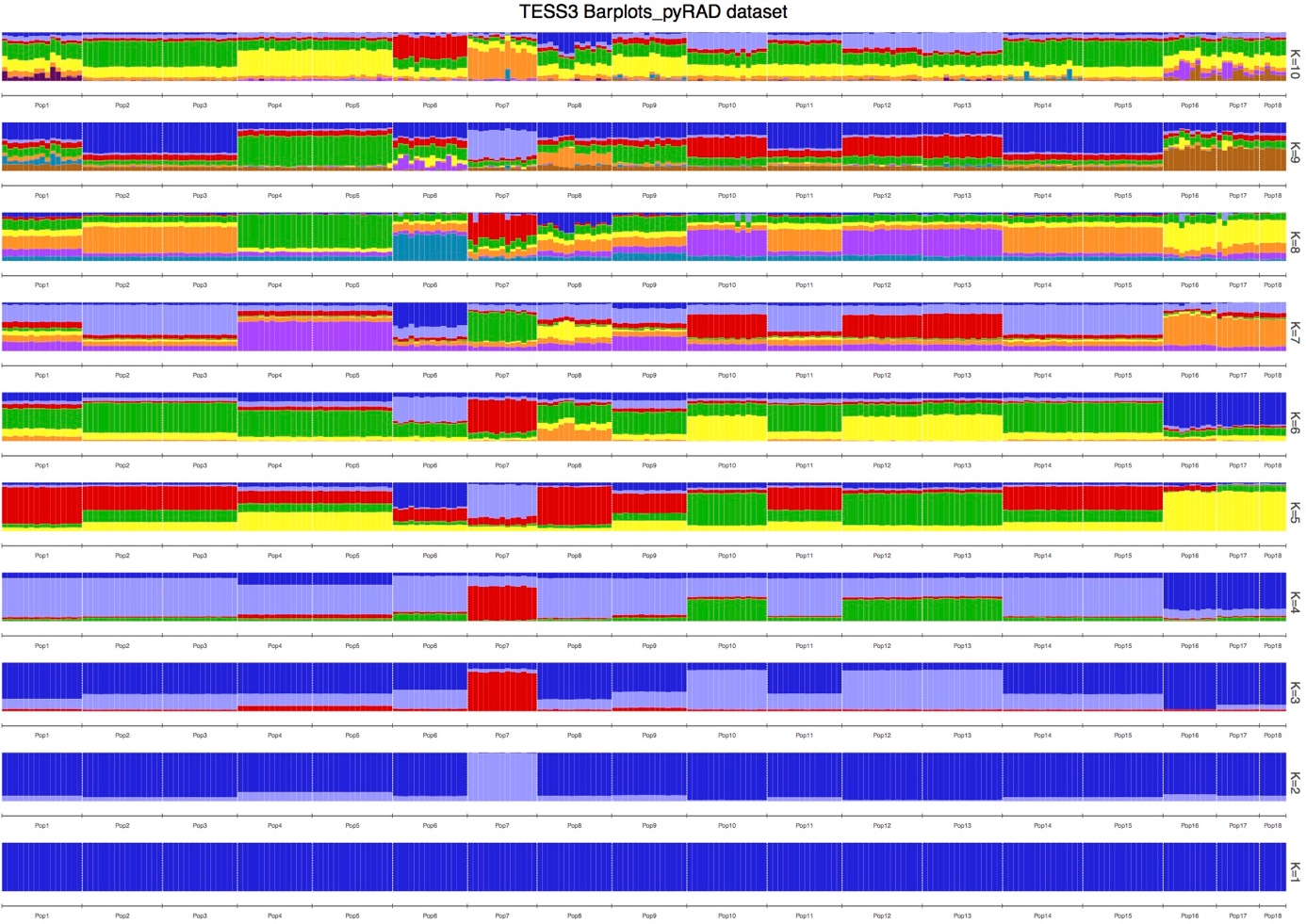


**Figure S3.** TESS3 clustering presented as barplots including 18 populations of *O. foetida* using pyRAD data set. The number of genetic clusters (K) are given on the right of each barplot, while the populations are indicated in below of each barplot and divided by white lines. (K=1 to K=10).


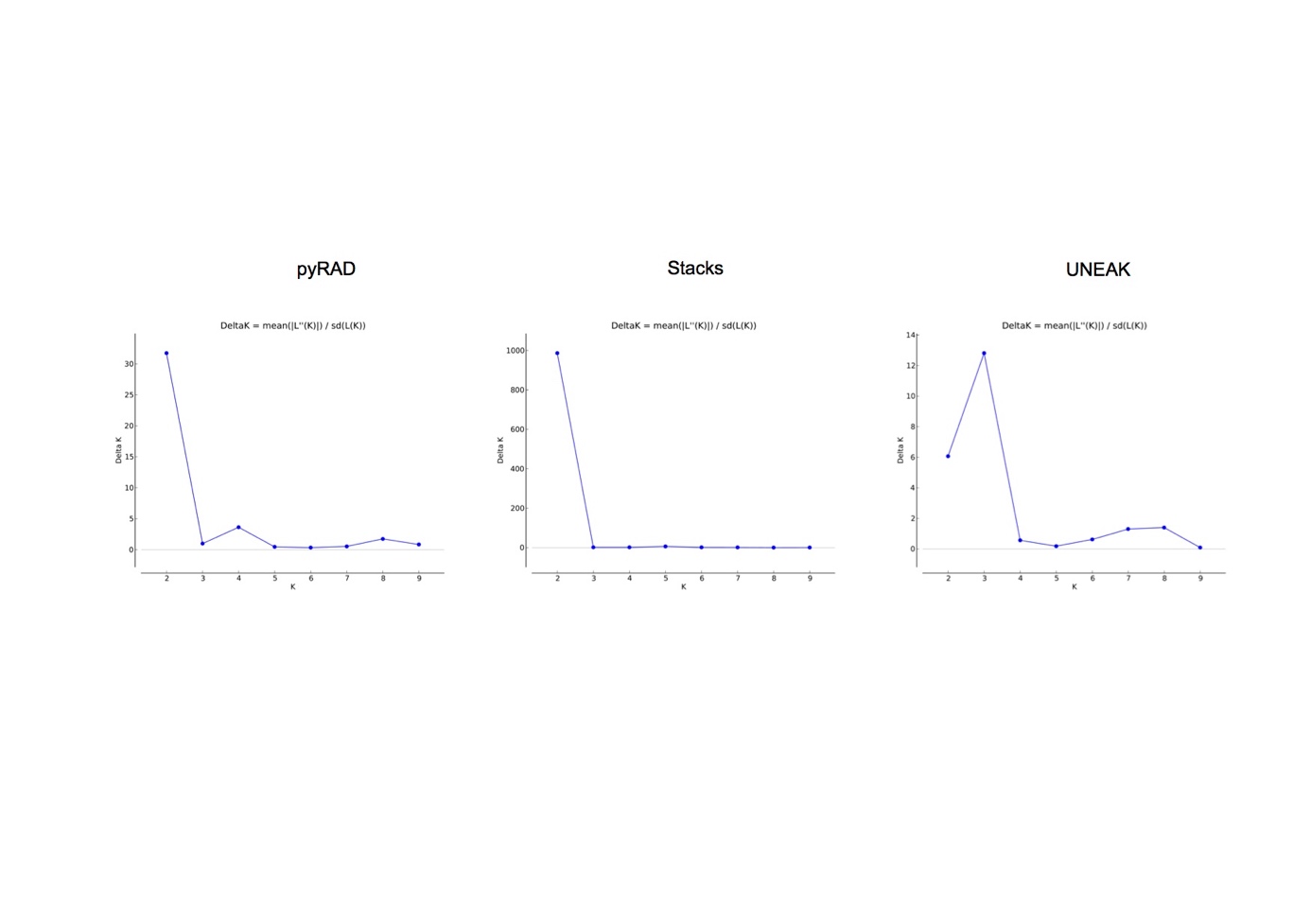


**Figure S4.** Delta K plots on (A)pyRAD, (B)Stacks and (C) UNEAK data sets


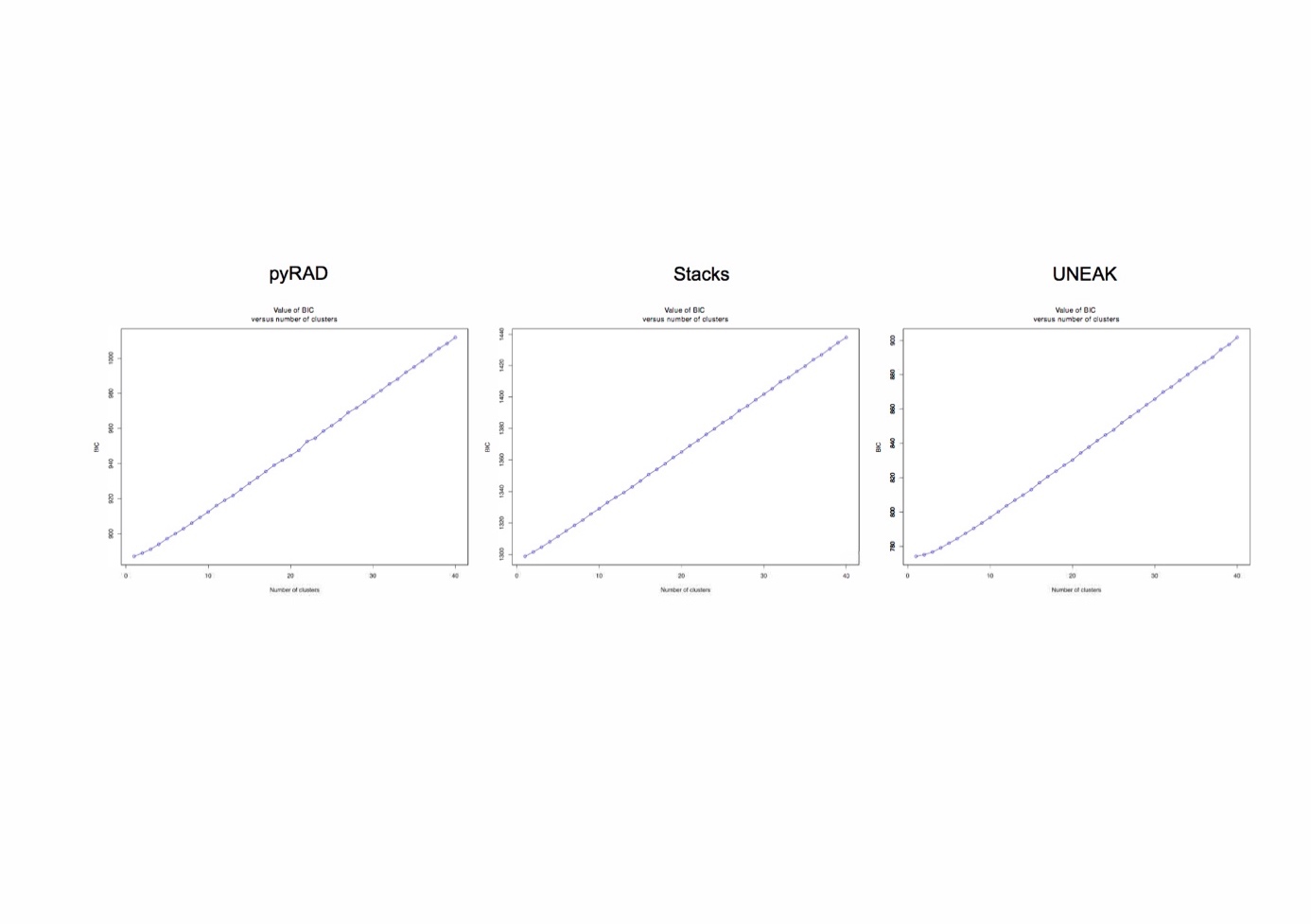


**Figure S5.** BIC plots for DAPC on (A)pyRAD, (B)Stacks and (C) UNEAK data sets
